# Do chief complaints allow targeting of SBIRT in the Emergency Department?

**DOI:** 10.1186/1940-0640-10-S2-O42

**Published:** 2015-09-24

**Authors:** Ryan McCormack, Phoebe Gauthier, Bridget McClure, Lauren Moy, Mei-Chen Hu, Martina Pavlicova, Edward V Nunes, David A Thompson, Raul Mandler, Michael Bogenschutz, Gail D'Onofrio, John Rotrosen

**Affiliations:** 1 Emergency Medicine, New York University School of Medicine, New York, 10016, USA; 2 Psychiatry, New York University School of Medicine, New York, 10016, USA; 3 Biostatistics, Mailman School of Public Health, Columbia University, New York, 10032, USA; 4 Psychitatry, Columbia University, New York, 10032, USA; 5 Emergency Medicine, Northwestern University School of Medicine, Illinois, 60611, USA; 6 National Institute on Drug Abuse, Maryland, 20852, USA; 7 Psychiatry, University of New Mexico School of Medicine, New Mexico, 87131, USA; 8 Emergency Medicine, Yale University School of Medicine, Connecticut, 06519, USA

## Background

Emergency Department (ED)-based Screening, Brief Interventions and Referral for Treatment (SBIRT) for alcohol and drug use has the potential to impact public health greatly. Time and resource constraints limit implementation [1]. Targeted intervention may be more efficient and practical. We hypothesized that we could use chief complaints to identify patients at highest risk of positive drug or alcohol use assessments.

## Material and methods

Using baseline data from NIDA CTN0047: SMART-ED [2], free text chief complaints of 14,972 subjects from six sites were coded using a tested algorithm [3]. Multiple team members manually reviewed and further collapsed the chief complaint categorization to ensure agreement. We excluded subjects having missing data or complaints related to substance use and chief complaints stated by <15 subjects. Positive screens were defined as AUDIT-C ≥4 for men and ≥3 for women (alcohol) and DAST ≥3 (drugs). We ranked-ordered the chief complaints by their sensitivity (i.e. greatest to fewest positive screens per complaint) and positive predictive value (i.e. proportion positive screens when the complaint is present) to 1) minimize the number of chief complaints and 2) assess the fewest number of ED patients. Our goal was to identify 75% of ED patients having positive assessments using these strategies.

## Results

**Figure 1:**
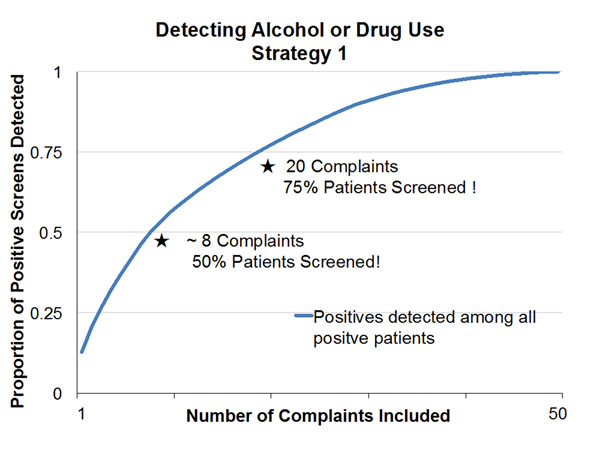


**Figure 2:**
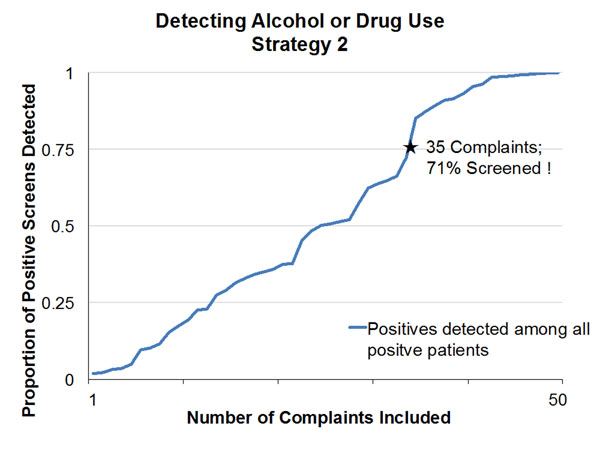


The screening assessments were positive in 5,805/14,561(39.9%) for alcohol and 2,454/14,494 (16.9%) for drugs. We collapsed the free-text chief complaints into 50 usable categories. To identify 75% of all ED patients having positive assessments using the first strategy would require including 19 chief complaints for alcohol screening and 20 chief complaints for drug screening. Adapting the second strategy, we would need to screen at least 71% and 68% of all ED patients for alcohol and drugs respectively to identify 75% of those having positive assessments. Among all ED patients screening positive for unhealthy alcohol or drug use. N=6,698 (46.0%), Figures 1 and 2 show the cumulative proportion of positive screens detected for each strategy when presenting complaints are used to select a subset of patients to screen. The horizontal axis adds complaints one at a time in descending order of complaint sensitivity in detecting positive screens (Strategy 1), and proportion positive screens when the complaint is present (Strategy 2).

## Conclusions

Based on this large, multicenter study, chief complaints provide little assistance in targeting SBIRT for alcohol or drug use in the ED.

## Trial Registration

NCT01207791

## Funding

NIDA CTN U10DA015833

NIDA CTN U10DA013035

NIAAA K23AA022989
